# Phalangeal Osteosarcoma Mistaken for Tuberculosis: A Case Report

**DOI:** 10.5704/MOJ.1503.011

**Published:** 2015-03

**Authors:** MA Mohd-Ariff, I Ali-Noor, AG Paul, S Abdullah

**Affiliations:** Department of Orthopaedics, Universiti Kebangsaan Malaysia Medical Centre, Kuala Lumpur, Malaysia

**Keywords:** Phalangeal osteosarcoma, osteosarcoma of the hand

## Abstract

We report a 21-year old female patient who presented with an 18-month history of a swelling in the distal phalanx of her right little finger. Although the history, clinical features and MRI were suggestive of a benign tumour or a tuberculous lesion, the histo-pathological examination of the swelling was reported as a conventional osteosarcoma. Osteosarcoma of the hand is very rare. This article highlights the possibility of a seemingly benign lesion seen in a routine clinic could well turn out to be malignant, and the need to maintain a high index of suspicion.

## Introduction

Osteosarcoma is the most common primary bone sarcoma typically originating from the metaphysis of long bones. It has a peak incidence at the age of 15-19 years with a high tendency for systemic spread^1^. The incidence of osteosarcoma of the hand has been reported to be between 0.18% – 0.39% of all skeletal osteosarcomas^2^. We report a rare presentation of osteosarcoma of the distal phalanx and its diagnosis and treatment.

## Case Report

A 21-year old Vietnamese female patient presented with an eighteen-month history of a right little finger swelling associated with increasing pain. She had no fever, loss of weight or loss of appetite.

**Fig. 1:**
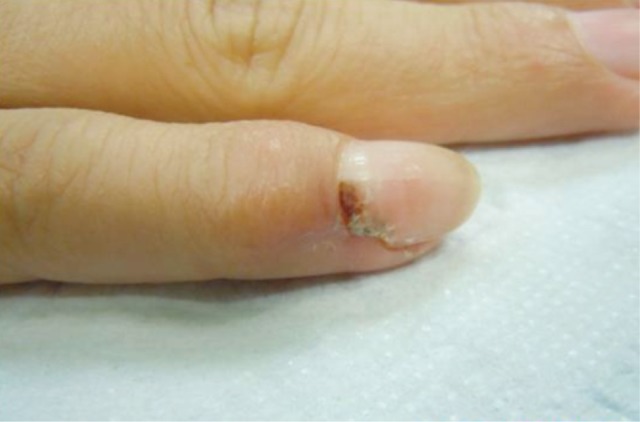
Painful right little finger swelling over the distal phalanx.

She had a small firm swelling measuring 1x1 cm^2^ on the dorso-lateral aspect of the distal phalanx of the right little finger (Fig. 1). It was tender to touch, with no signs of inflammation. Range of movement of the distal interphalangeal joint was normal. She had no epitrochlear or axillary lymphadenopathy.

**Fig. 2:**
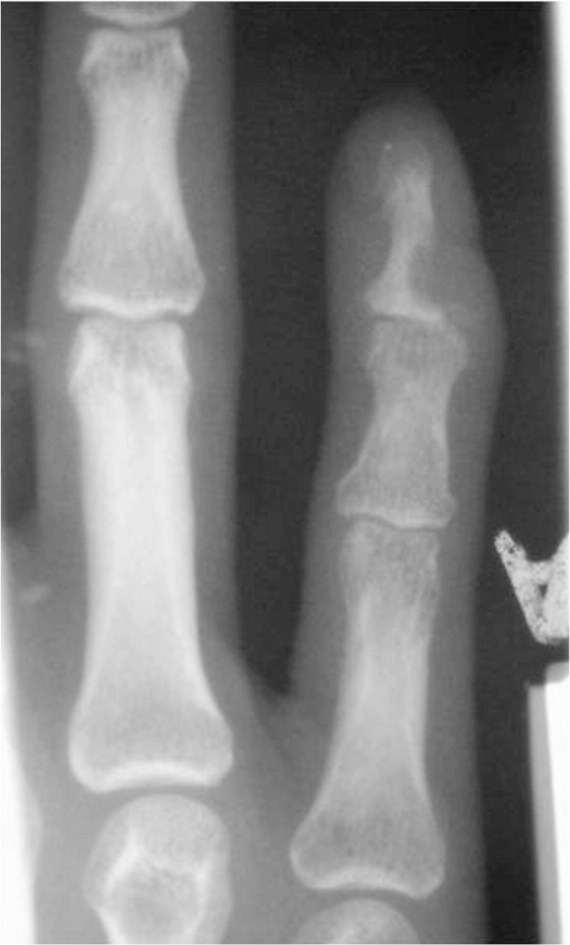
Plain radiograph with a well demarcated lytic lesion over the affected distal phalanx.

**Fig. 3:**
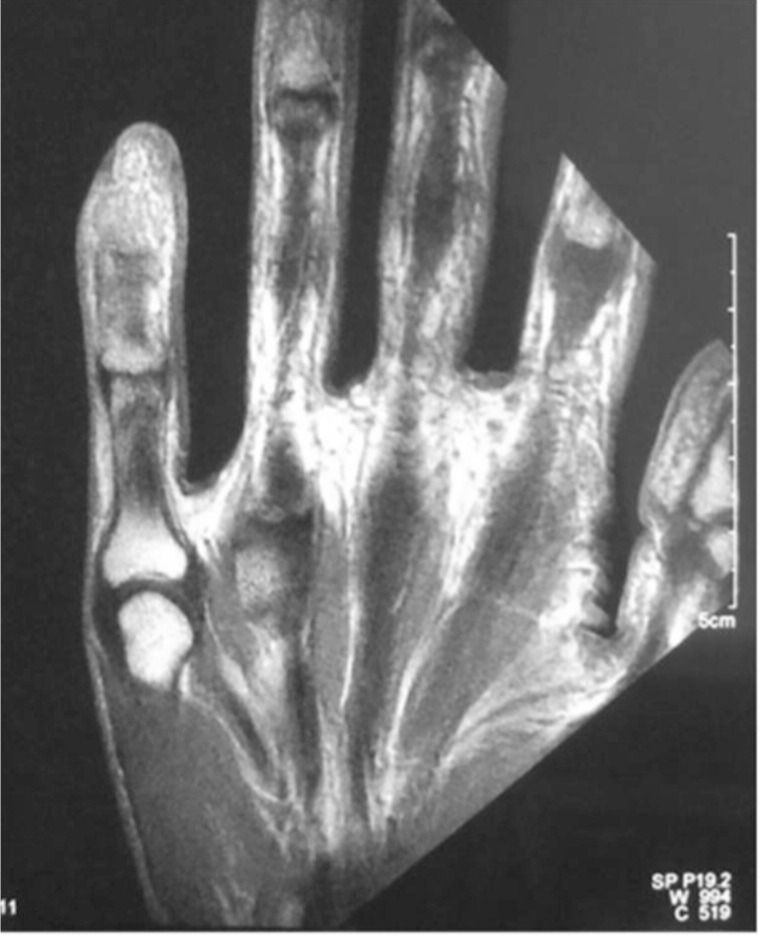
MRI revealing a lesion over the distal phalanx reported as a solid and enhancing osseous lesion with an extraosseous component with benign features, most likely tuberculosis. Histopathological examination, however, was reported as osteosarcoma.

Radiographs revealed a cystic lesion within the distal phalanx of the little finger (Fig. 2). MRI was reported as showing a solid and enhancing osseous lesion with an extraosseous component with benign features, most likely tuberculosis (Fig. 3). Chest radiographs were normal. Mantoux test was negative for active tuberculosis and ESR was 30mm/hr.

Our diagnosis of tuberculosis was based solely on the MRI findings as her history and physical findings were unremarkable.

**Fig. 4:**
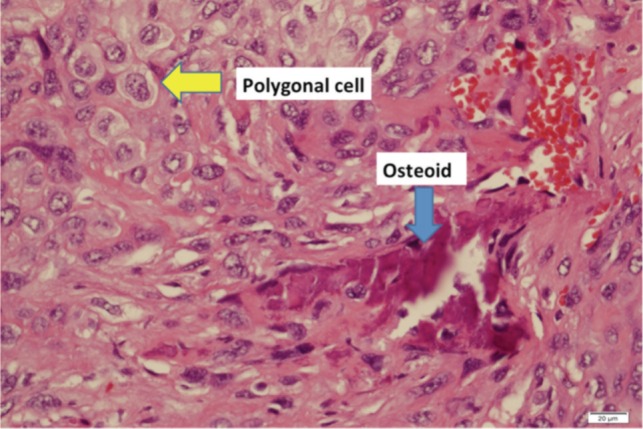
HPE slide showing osteoid formation seen (blue arrow) between malignant cells which are polyglonal and spindle shaped (yellow arrow).

Curettage was performed under local anaesthesia to confirm tuberculosis. However, to our surprise, the biopsy reported conventional osteosarcoma (Fig. 4). The biopsy section showed fragments of lesional tissue composed of a mixture of epithelioid cells, ovoid cells and spindle shaped cells with scattered non-neoplastic giant cells. Some tumor cells had hyperchromatic nuclei while others had vesicular nuclei with prominent nucleoli. A few mitotic figures were seen and some atypical forms were present. There was ample bone trabeculae with jagged edges and malignant lace-like osteoid deposition. No necrosis or granuloma was present.

A full physical examination did not reveal any possible primary tumours. The patient declined chemotherapy and a work-up for staging due to financial difficulties. We proceeded with amputation of the right little finger at the level of the middle phalanx. The histopathology report showed the margins to be clear with no residual tumor at the affected distal phalanx. Apart from a mild surgical site infection which resolved rapidly, she recovered uneventfully.

## Discussion

Fowble in 2005, reviewed 41 cases of osteosarcoma of the hand reported in 39 patients^3^ Eight of these cases had factors associated with osteosarcoma such as: Paget’s disease, radiation exposure and metastasis. Histologic evaluation was equivocal for the subtypes of osteosarcoma: fibroblastic,osteoblastic, chondroblastic, parosteal and periosteal. Age range was between 13 months and 85 years (average 43.3 years, median 40 years). Pain and swelling were the most common presentations similar to our patient. There was a predilection for the metacarpo-phalangeal joints with the second metacarpal head being the commonest site of involvement. This correlates with the most active bone growth site in the hand.

On the contrary, Kerin^4^ reviewed 123 cases of metastatic tumours of the hand and reported the more distal sites (i.e. terminal phalanges) were more often involved. Our patient’s presentation of idiopathic osteosarcoma arising solely in the distal phalanx is rare. There was a possibility that this could be a multifocal or multicentric osteosarcoma. However, our patient declined further investigations, hence we were unable to detect a possible primary tumour elsewhere. A bone scan in our patient may have allowed us to elucidate the cause of her terminal phalanx involvement.

There are no known associated patient risk factors for developing this rare tumour.

Conventional osteosarcoma is a grade by itself denoting a high-grade tumour.

The usual MRI findings in osteosarcoma of tubular bones are similar to those of long bones with aggressive lesions, cortical destruction and periosteal reaction, together with abnormal marrow signal intensity and peritumoral edema. It was the absence of these features in our patient’s MRI, which led us to a conclusion of a benign lesion. Osteosarcoma and tuberculosis have similar radiographic findings and extra vigilance with tuberculosis is in order as it is well known as the great mimic.

Evaluation and treatment of osteosarcoma of the phalanx should be similar to osteosarcoma of the long bones. Evaluation and treatment of conventional osteosarcoma of the fingers is amputation similar to long bones. Salvage was not an option as we had unknowingly performed curettage on a malignant lesion thus preventing us from obtaining clear tumour margins for such procedures. Chemotherapy and staging is indicated for osteosarcoma but the patient declined due to financial issues. Patients require CT evaluation of the thorax and abdomen and a bone scan, followed by the appropriate surgery and chemotherapy. Those with wide and radical margins had no local recurrence, but those with marginal excision had a considerably high recurrence rate^5^ In our patient, a definitive wide margin was achieved (amputation through the middle phalanx). Retrospectively, a high index of suspicion for osteosarcoma is needed for accurate diagnosis and prompt and proper treatment.

## Conclusion

We missed the diagnosis of osteosarcoma as our initial diagnosis of tuberculosis was based on the benign features of the patient’s physical examination findings and the MRI report and the increased risk of having come from a tuberculosis endemic area. A constant awareness for unusual lesions in unusual locations is important so that proper and prompt treatment can be provided for our patients.

## References

[1] Honoki K, Miyauchi Y, Yajima H, Takakura Y, Tamai S (2001). Primary osteogenic sarcoma of a finger proximal phalanx: a case report and literature review. J Hand Surg [Am].

[2] Okada K, Wold LE, Beabout JW, Shives TC (1993). Osteosarcoma of the hand: A clinicopathologic study of 12 cases. Cancer.

[3] Fowble VA, Pae R, Vitale A, Bryk E, Vigorita VJ (2005). Osteosarcoma of the hand.One case and a literature review. Clin Orthop Rel Res.

[4] Kerin R (1983). Metastatic tumors of the hand. A review of the literature. J Bone Joint Surg.

[b05] Amstutz HC (1969). Multiple osteogenic sarcomata: Metastatic or multi-centric?. Cancer.

